# Clinical trial landscape for guided bone regeneration: trend analysis and future perspectives

**DOI:** 10.3389/fdmed.2025.1689513

**Published:** 2025-10-27

**Authors:** Yuqing Gui, Xinyue Shen, Hongying Li, Junyi Lou, Junjie Cao, Jiang Yu, Zining Luo, Tianxiang Geng, Jiebin Xie

**Affiliations:** ^1^ Department of Gastrointestinal Surgery, Affiliated Hospital of North Sichuan Medical College, Nanchong, Sichuan, China; ^2^ North Sichuan Medical University, Nanchong, Sichuan, China; ^3^ Southwest Medical University, Luzhou, Sichuan, China; ^4^ Department of Orthopedics, Yantai Yuhuangding Hospital Affiliated with the Medical College of Qingdao University, Yantai, Shandong Province, China

**Keywords:** bone regeneration, bone graft, platelet-rich fibrin, synthetic bone substitutes, barrier membrane

## Abstract

**Aim:**

This study aims to comprehensively analyse the developmental progress of bone graft materials and barrier membranes in the field of oral-maxillofacial bone regeneration, with a particular emphasis on emerging therapeutic approaches for bone regeneration.

**Materials and methods:**

This study systematically searched 16 clinical trial registries using key terms such as “bone regeneration” and “osteoconduction” to identify relevant trials. The retrieved studies were then categorized and analysed on the basis of registration year, research phase, material/drug classification, and geographical distribution.

**Results:**

In the field of bone graft materials and barrier membranes, clinical trials involving synthetic bone graft materials (*N* = 90) and xenogeneic bone graft materials (*N* = 67) have been the most common. In the category of barrier membranes, collagen membranes still dominate (*N* = 53), whereas other natural membranes, such as amniotic and chorionic membranes, are in clinical trials. Resorbable polyester membranes (*N* = 24), titanium mesh (*N* = 13) and nonresorbable polytetrafluoroethylene membranes (*N* = 11) are commonly studied barrier membranes. Platelet-rich fibrin (PRF) (*N* = 71) is the most frequently used type of bioactive adjuvant. Some trials have explored the synergistic effects of statins (*N* = 17) and plant-derived active extracts (*N* = 16).

**Conclusion:**

Research on bone regeneration is undergoing a paradigm shift from the conventional “bone graft + barrier membrane” approach to integrated multicomponent strategies. These advanced strategies combine tunable biodegradable scaffolds, growth factors, and small-molecule drugs to achieve personalized and cost-effective bone defect repair. Future research priorities will focus on optimizing material degradation kinetics and spatial maintenance properties to enhance clinical outcomes.

## Introduction

Tooth loss is a common oral disease. It is estimated that in the United States, two out of every three individuals have at least one missing tooth. Annually, over 15 million people undergo dental implant placement as a replacement therapy (1). Prior to implant placement, bone regeneration treatment is often required to ensure sufficient bone volume in the deficient area; otherwise, the undesirable consequence of reduced implant stability may occur. Alveolar ridge atrophy caused by tooth loss or alveolar bone resorption following tooth extraction often results in insufficient bone volume needed for implant placement. Moreover, maxillary sinus expansion frequently accompanies the extraction of posterior maxillary teeth, affecting the residual alveolar ridge area. Therefore, the alveolar ridge and the maxillary sinus floor are essential sites for bone regeneration treatment (2, 3).

Bone regeneration treatment often requires bone graft materials. Autografts are considered the “gold standard” for clinical bone graft materials and the most effective method for bone regeneration. They promote new bone formation on the bone surface through direct osteoconduction and induce the differentiation of local stem cells into osteoblasts without causing any immune rejection reactions. However, autografts also face issues such as donor site trauma and limited availability (4). Owing to their wide availability and ease of acquisition, xenografts have become the most widely used bone substitutes for peri-implant and periodontal regeneration applications (5). A study comparing autografts and porcine xenografts for maxillary sinus floor augmentation reported similar bone regeneration outcomes. However, they provide only a scaffold and do not promote bone formation by themselves (6). Owing to their osteoconductive and osteoinductive properties, allografts are considered alternatives to autografts. However, due to increased risks of host immune reactions and disease transmission, their use has decreased. Synthetic bone substitutes, which closely resemble the inorganic components of bone and are resorbable, are considered promising alternatives to autograft materials (7).

Bone grafts are often combined with barrier membranes to achieve guided bone regeneration (GBR). The biological basis lies in the “cell exclusion principle”: the sequential occupation of the regenerative space by different cell types affects the type of tissue that eventually forms. Barrier membranes physically exclude nonosteogenic cells (such as fibroblasts) from entering the defect area, thereby preserving the space for bone regeneration and supporting the migration of bone-related cells and bone tissue formation (5). Many studies have focused on natural resorbable collagen membranes as well as synthetic membranes, including polylactic acid (PLA), polyglycolic acid (PGA), poly(*ε*-caprolactone) (PCL), and their copolymers and derivatives.

In addition to traditional bone substitute materials, bioactive adjuvants have recently garnered significant attention. Bioactive factors such as platelet-rich fibrin (PRF), statins, and some plant-derived active extracts have shown potential for promoting bone regeneration in animal experiments and early clinical studies.

This study systematically reviews clinical trials conducted in the field of oral and maxillofacial bone regeneration up to the year 2025. By descriptively analysing the present application landscape of bone regeneration materials, this research seeks to provide a basis for policymakers to rationally allocate funding for bone regeneration materials and to offer new directions for the development of bone graft materials and barrier membranes, as well as for the study of pharmaceuticals and bioactive factors that promote bone regeneration.

## Methods

**Figure 1:**
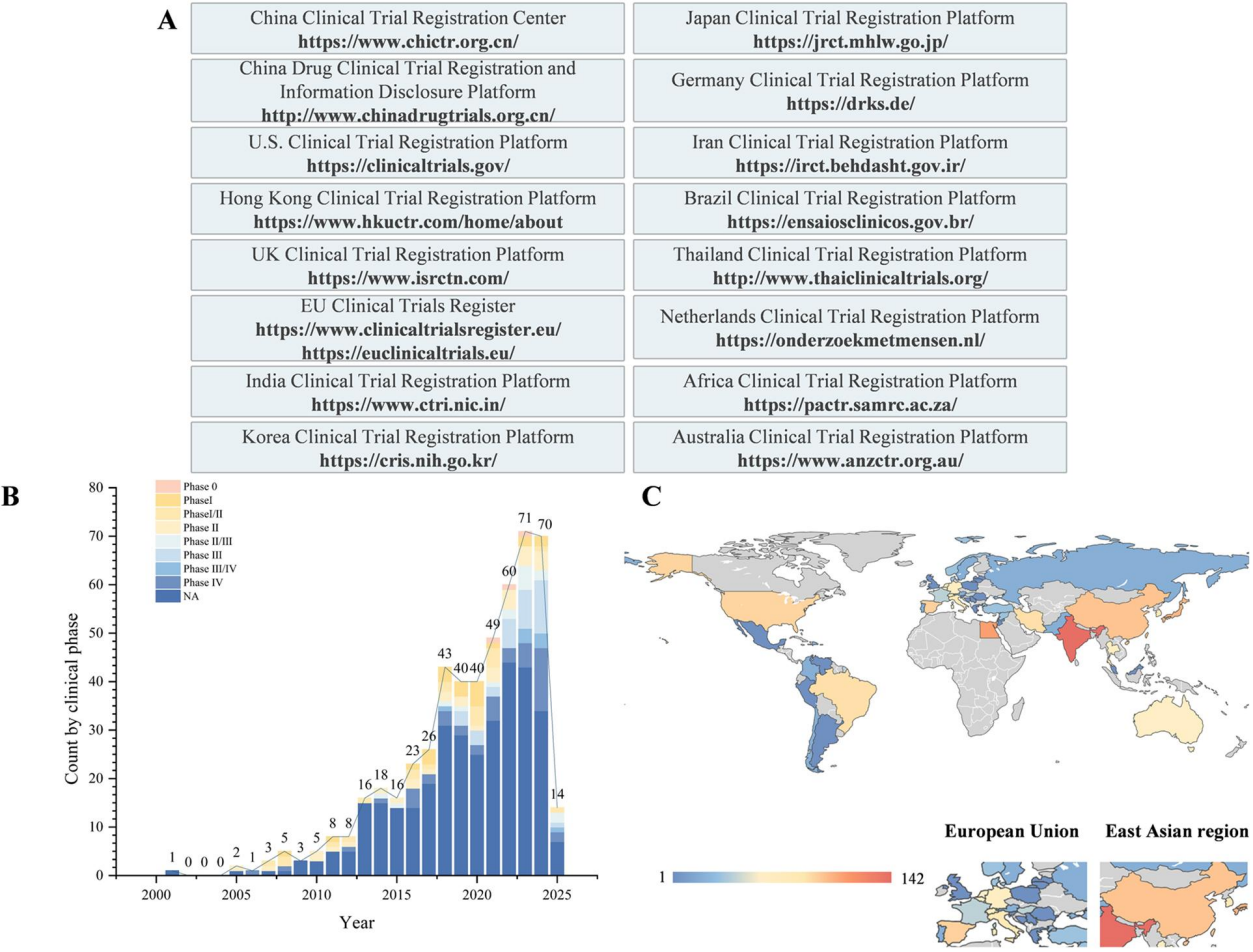
Overview of bone regeneration research: materials, databases, and geographical distribution of clinical trials. **(A)** Clinical trial databases and their URLs. **(B)** Temporal and clinical phase distributions of clinical trials on bone regeneration. **(C)** Geographical distribution of clinical trials on bone regeneration.

This study systematically analysed the current landscape of clinical trials related to bone regeneration by searching 16 clinical trial registries up to March 20, 2025 (Figure 1A). Using professional terms from the PubMed MeSH and Embase Emtree systems, we constructed search queries that included “bone regeneration”, “eneration, bone”, “regeneration, bone” and “osteoconduction” along with their synonymous extensions. A total of 787 clinical trials were retrieved from 16 clinical trial registries. Among these, studies specifically focusing on materials, pharmaceuticals, bioactive factors, or techniques aimed at promoting oral and maxillofacial bone regeneration were included. After excluding duplicate registrations, trials unrelated to bone regeneration, and those with unclear objectives, 522 clinical trials were ultimately included in the analysis (Supplementary Table S1). The core elements extracted and compared included the year of registration and trial phase; types of bone graft materials used (autografts, allografts, xenografts, and synthetic substitutes); categories of barrier membranes, growth factors or drugs; and geographical regions where the trials were conducted. All the statistical analyses were performed via Excel. Categorical variables are summarized by frequency and are presented as counts (percentages). Data visualization was conducted in Origin 2021, with bar charts, sankey charts, etc., generated according to the data type. Python 3.12 was used to draw the geographic distribution map of the clinical trials. All the analytical procedures were independently performed by two investigators and cross-validated to ensure accuracy.

## Results

A total of 787 clinical trials were identified. After excluding duplicate records and trials unrelated to the topic, 522 clinical trials were ultimately included. Temporal trend analysis revealed a significant increase in the number of related clinical trials starting in 2018, with an average of approximately 53.3 trials per year (Figure 1B).

### Bone graft material

**Figure 2:**
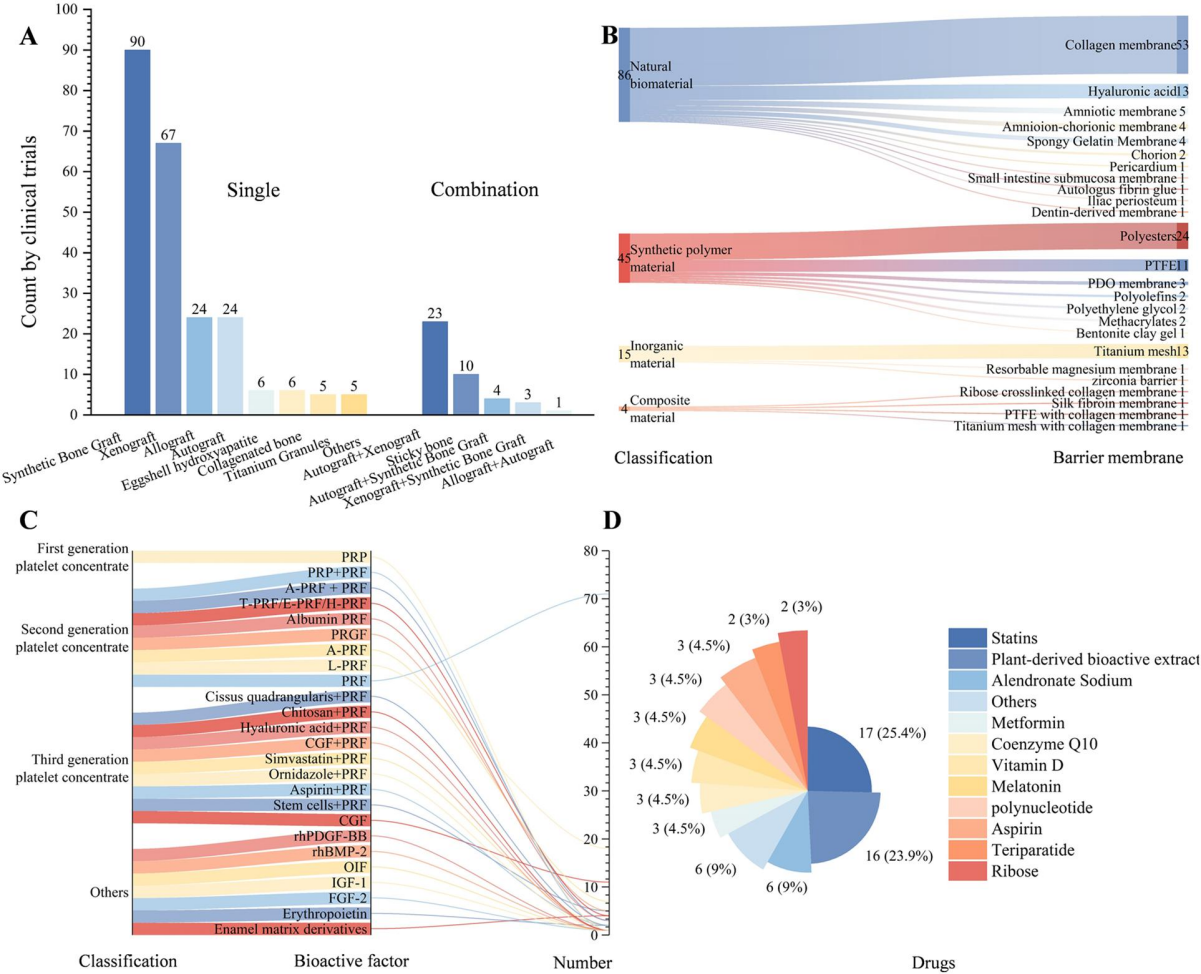
Characteristics and distribution of clinical trials in bone regeneration research. **(A)** Types of bone graft materials and the corresponding number of trials; **(B)** Types of barrier membranes and the corresponding number of trials; **(C)** Types of bioactive factors and the corresponding number of trials; **(D)** Types of drugs and the corresponding number of trials (natural extracts include Cissus quadrangularis, curcumin, asigannan, acemannan, mangosteen peel extract gel, astaxanthin, grape seed and grapefruit seed extract, Morinda citrifolia fruit extract, oleuropein, psidium guajava leaf and trans-resveratrol).

Synthetic bone substitutes (including hydroxyapatite, *β*-tricalcium phosphate, biphasic calcium phosphate, or bioactive glass) were the most frequently used (*n* = 90), followed by xenografts (*n* = 67). In recent years, several novel materials, such as eggshell hydroxyapatite and collagen-based bone materials, have emerged. Combinations of xenografts with autografts (*n* = 23) and the clinical validation of “sticky bone” mixtures prepared with PRF and bone graft materials (*n* = 10) have also been reported (Figure 2A).

### Barrier membranes

Among naturally derived barrier membranes, collagen membranes remained the predominant choice (*n* = 53). Hyaluronic acid (HA) (*n* = 13) is the second most common. Amniotic and chorionic membranes have entered early clinical evaluation. Resorbable polyester membranes (e.g., PCL, PLA, and PLGA; *n* = 24), titanium mesh (*n* = 13), and nonresorbable polytetrafluoroethylene (PTFE) membranes (*n* = 11) continue to be common research subjects (Figure 2B).

### Bioactive factors and drugs

The second-generation platelet concentrate, PRF (*n* = 71), is the most commonly used bioactive factor. Third-generation formulations, concentrated growth factor (CGF), and combinations of multiple drugs with PRF have attracted increasing research interest (Figure 2C). In terms of pharmacological interventions, statins (*n* = 17) and alendronate (*n* = 6) are the most frequently studied. Additionally, various plant-derived active extracts (*n* = 16), such as curcumin, guava leaf extract, mangosteen rind, and grape (or pomelo) seed extracts, are being explored for their potential to enhance bone regeneration (Figure 2D).

## Discussion

This study provides a systematic analysis of the current state of clinical research and development in oral and maxillofacial bone regeneration. Since 2018, the number of studies on bone regeneration has significantly increased, with a growing trend annually. Synthetic bone substitutes have surpassed xenogeneic bone grafts as the most frequently investigated bone graft materials. While traditional collagen membranes remain predominant, research on hyaluronic acid, absorbable polyester membranes, and modified collagen membranes has gradually increased. Among the bioactive factors, PRF is currently the most extensively studied platelet-derived product and is often combined with bone graft materials to form sticky bone. Additionally, few studies have explored the potential of statins, bisphosphonates, and plant-derived extracts in promoting bone regeneration.

Synthetic bone substitutes, including hydroxyapatite (HA), β-tricalcium phosphate (β-TCP), biphasic calcium phosphate (BCP), and bioactive glass (BAG), have garnered widespread clinical interest because of their ability to be customized into regenerative scaffolds with sufficient mechanical strength and patient-specific morphology for bone defects through precise control of pore size, density, and 3D printing. Owing to their inert ceramic nature, they typically do not elicit foreign body reactions (6). However, a significant limitation of synthetic grafts is their lack of bioactive elements—such as Ti, Cu, Co, Sr, Li, Mo, and Zn—found in natural bone, which may contribute to their suboptimal clinical performance. Consequently, modification with inorganic ions has been recognized as a promising strategy to increase the biological efficacy of bone-centered biomaterials (8). For example, copper ions, known for their proangiogenic and antibacterial properties, have demonstrated promising results in promoting bone regeneration in the context of periodontal infection (9). This approach has the potential to overcome the limitations of conventional treatment protocols following tooth extraction due to severe caries, periapical periodontitis, or periodontal disease—which often require weeks or months of infection control prior to bone grafting—thereby significantly shortening the treatment timeline and effectively preventing postextraction alveolar ridge resorption. On the other hand, the absorption rate of synthetic bone substitutes is often difficult to control, frequently resulting in either excessively rapid absorption—which compromises the maintenance of the defect space—or overly slow absorption, leading to long-term risks of infection associated with foreign body reactions. The study by Koichiro Hayashi et al. addressed this challenge through the rational design of a hierarchical pore structure (comprising macropores, micropores, and nanopores), enabling the material to degrade in a timely manner within four weeks while being replaced by newly formed bone (9). Optimizing the pore size distribution presents a feasible reference solution for balancing absorption control and mechanical strength in synthetic bone substitutes.

Compared with natural barrier membranes such as collagen and hyaluronic acid, synthetic polyester membranes (e.g., PCL, PLA, and PLGA) exhibit superior mechanical properties. They also offer low immunogenicity and tunable degradation rates and have been proven to effectively maintain the osteogenic space. However, a major challenge for most synthetic polymer membranes lies in their insufficient cell adhesion properties. To address this, these synthetic polymers are often blended with natural polymers (10). For example, a recent study incorporated a PLA-based composite with hyaluronic acid and further enriched it with 30% β-TCP. This modification not only enhanced the mechanical performance of the material but also effectively combined the barrier function of the membrane with the osteogenic capability of the synthetic bone graft, resulting in an increased bone formation rate of 28.9%. In comparison, a rate of 24.9% was observed with the collagen membrane Epi-Guide® (11). Compared with natural barrier membranes, such hybrid membranes—with greater ductility and mechanical strength—reduce the technical sensitivity associated with suturing the membrane to the wound site during bone regeneration procedures.

Platelet-derived preparations, especially PRF, which contain high concentrations of platelets, leukocytes, and growth factors such as TGF-β1, PDGF, and VEGF, have demonstrated superior antimicrobial and osteogenic dual effects in deep periodontal pockets and high-risk infection environments (12). Meta-analyses have shown that PRF, whether used alone or in combination with bone graft materials, statins, metformin, or bisphosphonates, can significantly increase new bone volume (13). Third-generation platelet concentrates are further expanding their indications and efficacy by being combined with drugs or bioactive ceramics.

Plant-derived active extracts have garnered attention because of their combined antioxidant, antimicrobial, and immunomodulatory activities. Mangosteen rinds, which are rich in mangostin, can enhance the differentiation of mesenchymal stem cells into the osteogenic lineage and reduce osteoclast activity (14). Cissus quadrangularis and grape seeds have also been shown to potentially regulate the osteogenesis/osteoclastogenesis balance (15, 16). These low-toxicity natural molecules may serve as cost-friendly adjuvants that can be used in synergy with existing bone substitutes.

Compared with single-material approaches, bone tissue engineering, which combines stem cells, growth factors, and bone scaffolds, holds promise for reshaping the landscape of bone regeneration (6). In addition to the use of collagen membranes, hyaluronic acid, or synthetic membranes to encapsulate stem cells or deliver plasmid DNA, which promotes bone formation and controls the delivery of growth factors, increasing attention has been given to placenta-derived membranes, namely, the amnion (AM) and chorion (CM). These materials are considered ideal natural scaffolds for bone tissue engineering because of their low immunogenicity and abundance of matrix proteins, stem cells, and endogenous growth factors. The epithelial/mesenchymal cells of AM exhibit multipotent differentiation potential, and their secretion of anti-inflammatory factors promotes aseptic healing. CM, which is three times thicker than AM, has been shown *in vitro* to significantly increase the expression of osteogenesis-related genes and increase calcium deposition levels (with increases of approximately 25-fold and 2- to 3-fold in calcium ions, respectively) (17, 18). Leveraging the “surgical waste” nature of placental tissue, these membranes face no ethical barriers and are abundantly available, presenting significant potential for large-scale applications (2). Furthermore, as single-function cell implants have become insufficient to meet the demands of advanced tissue engineering, nanohybrid technology represents another breakthrough in the field. Recent studies have hybridized Prussian blue nanoparticles (PBNPs) with various cell types through self-organization to form multicellular spheroids (MCSs). The resulting PBNP-hybridized multicellular spheroids (PBNPs@MCSs) exhibit enhanced antioxidant functionality, effectively reducing apoptosis under oxidative stress conditions. This significantly improves cell survival duration and rates in inflammatory oral environments and enhances the capacity to promote bone repair (19). This innovative strategy may offer new therapeutic options for patients experiencing alveolar bone resorption following tooth extraction, ultimately accelerating alveolar bone healing.

This study is a clinical landscape analysis that fundamentally involves the secondary analysis and synthesis of registered clinical trials rather than prospective original research. As such, our findings and conclusions are highly dependent on the quality, completeness, and reporting standards of the original trial records. Although every effort has been made to analyse current clinical trials in bone regeneration comprehensively, the lack of publicly accessible clinical databases in certain regions limits our ability to obtain a more complete dataset. Furthermore, despite the implementation of stringent inclusion and exclusion criteria and independent screening by two investigators, a degree of subjectivity remained unavoidable in assessing the eligibility of certain studies. Notably, landscape analysis aims to depict the current state and emerging trends within the field and does not involve evaluating the efficacy of various materials in promoting bone regeneration. Future rigorously designed and standardized controlled studies will be essential to validate the true therapeutic effectiveness of different regenerative materials.

In conclusion, bone regeneration strategies are transitioning from the “material + barrier membrane” paradigm to an era of “multicomponent integration”: programmable degradable scaffolds, immune-regulating molecules, targeted release of growth factors, and personalized 3D-printed structures will work together to achieve precise regeneration. Moreover, the integrated repair of bone and soft tissue and the regulation of the inflammatory microenvironment are frontiers that urgently need breakthroughs. By optimizing both material microstructure design and biological signal modulation, efficient, safe, and personalized bone defect repair solutions can be achieved while reducing economic burdens.

## Data Availability

The original contributions presented in the study are included in the article/Supplementary Material, further inquiries can be directed to the corresponding author.
