# Supplementary material for: Clinical trial landscape for guided bone regeneration: trend analysis and future perspectives
Source: Front Dent Med. 2025 Oct 27;6:1689513. doi: 10.3389/fdmed.2025.1689513 (PMC12598897; doi:10.3389/fdmed.2025.1689513)
Supplement: Supplementary file 1 [file Table1.docx]

**Clinical Trial Landscape for Guided Bone Regeneration: Trend analysis and future perspectives**

**Authors**

Yuqing Gui^1,2^**^*^**, Xinyue Shen^3^**^*^**, Hongying Li^2^**^*^**, Junyi Lou^2^**^*^**, Junjie Cao^2^, Jiang Yu^2^, Zining Luo^2^**^†^**, Tianxiang Geng^4^**^†^**, Jiebin Xie^1^**^†^**

^* These authors contributed equally to this article.^

**^†^** ^These authors contributed equally to this article and should be recognized as the corresponding authors.^

**Corresponding Author**

Zining Luo

Tianxiang Geng

Jiebin Xie

^1^Department of Gastrointestinal Surgery, Affiliated Hospital of North Sichuan Medical College

^2^North Sichuan Medical University, Nanchong, Sichuan, China.

^3^Southwest Medical University, Luzhou, Sichuan, China.

^4^Department of Orthopedics, Yantai Yuhuangding Hospital Affiliated with the Medical College of Qingdao University, Yantai, 264000, Shandong Province, China

^1^Affiliated Hospital of North Sichuan Medical University, Nanchong, Sichuan, China

**Acknowledgments**

We would like to extend our gratitude to the developers of the plotly library for providing the world map templates used in this study. We used the built-in map template. We respect the territorial integrity and sovereignty of all nations, and the national boundaries as displayed by these database templates do not represent the authors' political stance.

**Funding**

The author(s) declare that financial support was received for the research and/or publication of this article. This study was supported and funded by the National Natural Science Foundation of China (81702093), the Youth Program of the Department of Education (13ZB0243), the Sichuan Medical Association (SC202401), the Sichuan Medical Association (2024HR09), Science and Technology Development Plan of the Affiliated Hospital of North Sichuan Medical College (2022LC005) and the Youth Program of the Open Fund of the State Key Laboratory of Innovative Drug Research for Neurological and Oncological Disorders (SKLSIM-F-202443).

**Authors' contributions**

YG: Conceptualization, Formal analysis, Methodology, Writing – original draft, Writing – review & editing. XS: Data curation, Formal analysis, Methodology, Writing – review & editing. HL: Data curation, Formal analysis, Methodology, Writing – review & editing. JL: Data curation, Formal analysis, Methodology, Writing – original draft. JC: Data curation, Formal analysis, Methodology, Writing – original draft, Writing – review & editing. JY: Data curation, Formal analysis, Methodology, Writing – review & editing. ZL: Funding acquisition, Project administration, Supervision, Writing – review & editing. TG: Funding acquisition, Project administration, Supervision, Writing – review & editing. JX: Funding acquisition, Project administration, Supervision, Writing – review & editing.

**Ethics approval and consent to participate**

Not applicable.

**Consent for publication**

Not applicable.

**Data availability statement**

The original contributions presented in the study are included in the article/Supplementary Material, further inquiries can be directed to the corresponding author. **Conflict of interest**

The authors declare that the research was conducted in the absence of any commercial or financial relationships that could be construed as a potential con0ict of interest.

**Catalogue**

[**Table 1**: Characteristics of clinical trials for bone regeneration 3](#_Toc195446669)

**Table 1**: **Characteristics of clinical trials for bone regeneration**

| **Registration number** | **Year** | **Nation** | **Trial phase** | **Bone graft material** | **Barrier membrane** | **Others** | **Sample size** |
| --- | --- | --- | --- | --- | --- | --- | --- |
| NCT06842368 | 2025 | Egypt | NA | Xenograft | NA | Hyaluronic acid+Vitamin D | 24 |
| NCT06831214 | 2025 | Egypt | NA | NA | Iliac periosteum | Sausage technique | 22 |
| NCT06827665 | 2025 | Jordan | Phase Ⅰ/Ⅱ | Allograft | NA | NA | 10 |
| NCT06746935 | 2025 | Egypt | NA | NA | PTFE | NA | 20 |
| NCT06732167 | 2025 | Spain | NA | Xenograft | NA | NA | 68 |
| jRCT1051240285 | 2025 | Japan | NA | NA | NA | Mesenchymal stem cells | NA |
| CTRI/2025/03/081739 | 2025 | India | NA | NA | NA | L-PRF+Aspirin | 20 |
| CTRI/2025/02/081201 | 2025 | India | Phase Ⅳ | NA | NA | PRP | 20 |
| CTRI/2025/02/080695 | 2025 | India | Phase Ⅲ/Ⅳ | Autograft | NA | PRF | 20 |
| CTRI/2025/01/079533 | 2025 | India | Phase Ⅱ/Ⅲ | NA | Collagen membrane | PRF+Cissus quadrangularis | 30 |
| CTRI/2025/01/079226 | 2025 | India | Phase Ⅲ | Allograft | NA | PRF | 20 |
| CTRI/2025/01/078984 | 2025 | India | Phase Ⅳ | Bone putty | NA | PRF | 44 |
| CTRI/2025/01/078937 | 2025 | India | Phase Ⅱ/Ⅲ | Autograft+Xenograft | TR-d-PTFE | NA | 12 |
| ACTRN12625000122459 | 2025 | Australia | NA | NA | Resorbable polycaprolactone network | 3d-printed | 24 |
| TCTR20241002007 | 2024 | Thailand | Phase Ⅲ | Xenograft | NA | Autologous blood/PRF | 45 |
| RBR-7tkkqj4 | 2024 | Brazil | NA | RAPG | NA | NA | 20 |
| RBR-7q7vw68 | 2024 | Brazil | NA | Autologous dentin | NA | NA | 10 |
| NCT06773923 | 2024 | Chile | Phase Ⅲ | Synthetic bone substitute material | PCL | Metformin+3d-printed Resorbable Scaffolds | 10 |
| NCT06721806 | 2024 | NA | NA | NA | NA | PRP | 42 |
| NCT06695338 | 2024 | Spain | NA | NA | NA | Guided Bone Regeneration (Submerged) Guided Bone Regeneration (Non-submerged) | 32 |
| NCT06692244 | 2024 | China | NA | NA | Titanium meshes | NA | 142 |
| NCT06662604 | 2024 | NA | Phase Ⅲ | NA | NA | Bone marrow stem cells+PRF | 22 |
| NCT06657651 | 2024 | Egypt | NA | NA | Dentin-derived membrane | PRF | 40 |
| NCT06636084 | 2024 | United States | NA | NA | TR-PTFE | NA | 32 |
| NCT06633211 | 2024 | Spain | NA | Xenograft | NA | polynucleotide+hyaluronic acid | 20 |
| NCT06570265 | 2024 | NA | NA | NA | Collagen membrane | NA | 50 |
| NCT06559605 | 2024 | Egypt | NA | NA | Zirconia barrier Titanium mesh | 3d-printed | 26 |
| NCT06552936 | 2024 | Pakistan | NA | NA | NA | Diode Laser 980 nm. | 44 |
| NCT06552715 | 2024 | Italy | Phase Ⅰ | Synthetic Bone Graft | NA | NA | 20 |
| NCT06552065 | 2024 | India | NA | NA | Silk fibroin membrane | NA | 20 |
| NCT06513039 | 2024 | Egypt | Phase Ⅳ | NA | NA | Erythropoietin+Chitosan | 15 |
| NCT06452134 | 2024 | NA | Phase Ⅳ | NA | NA | Coenzyme Q10 | 70 |
| NCT06383377 | 2024 | Belgium | NA | Xenograft | NA | NA | 96 |
| NCT06338241 | 2024 | Spain | Phase Ⅳ | Synthetic Bone Graft | NA | Simvastatin | 30 |
| NCT06295055 | 2024 | Thailand | NA | NA | Semi-rigid shell barrier system | NA | 10 |
| NCT06286605 | 2024 | Italy | NA | Autograft+Xenograft | NA | NA | 42 |
| NCT06257225 | 2024 | NA | NA | NA | Collagen membrane | NA | 22 |
| NCT06247098 | 2024 | United States | NA | Autograft+Xenograft | NA | NA | 10 |
| NCT06242782 | 2024 | NA | Phase Ⅲ | NA | d-PTFE e-PTFE RPM（Resorbable Polymeric Membrane） Titanium mesh | NA | 148 |
| NCT06141239 | 2024 | Taiwan | NA | Autologous Tooth Root + bone graft | NA | Vit D | 48 |
| KCT0009560 | 2024 | Korea | NA | Sticky bone | NA | NA | 40 |
| KCT0009145 | 2024 | Korea | Phase Ⅰ | ceramic scaffold | NA | Polydeoxyribonucleotide+3d-printed ceramic scaffold | 6 |
| jRCTs072240085 | 2024 | Japan | Phase Ⅱ | Synthetic bone substitute material | NA | NA | 10 |
| jRCTs032240389 | 2024 | Japan | NA | Synthetic Bone Graft | NA | NA | 10 |
| jRCTs032240332 | 2024 | Japan | NA | NA | P（LA/CL）membrane | NA | 10 |
| jRCTb030240452 | 2024 | Japan | NA | Autograft | NA | Adipose-derived stem cells+PRF | 4 |
| IRCT20240721062500N1 | 2024 | Iran (Islamic Republic of) | NA | NA | NA | Low level laser therapy | 17 |
| IRCT20130813014350N6 | 2024 | Iran (Islamic Republic of) | Phase Ⅲ | Sticky bone/xenograft | NA | NA | 10 |
| DRKS00036122 | 2024 | Germany | NA | Allograft/Xenograft/Synthetic bone substitute material | NA | PRF | 180 |
| DRKS00032281 | 2024 | Serbia Switzerland | NA | NA | NA | Arm 1：Immediate implant placement (IIP) Arm 2：Alveolar ridge preservation (ARP) followed by Implant placement | 50 |
| CTRI/2024/12/078652 | 2024 | India | Phase Ⅲ | Xenograft | NA | PRF | 20 |
| CTRI/2024/12/078041 | 2024 | India | Phase Ⅱ | NA | NA | PRF | 10 |
| CTRI/2024/11/077308 | 2024 | India | Phase Ⅳ | NA | NA | Enamel matrix derivatives | 30 |
| CTRI/2024/11/077158 | 2024 | India | Phase Ⅳ | NA | PLGA scaffold | 3d-printed | 10 |
| CTRI/2024/11/076550 | 2024 | India | Phase Ⅱ | Xenograft | NA | NA | 30 |
| CTRI/2024/10/074804 | 2024 | India | Phase Ⅲ/Ⅳ | NA | NA | PRF/Simvastatin | 40 |
| CTRI/2024/10/074763 | 2024 | India | NA | Autograft+Xenograft | Collagen membrane/Titanium mesh | NA | 18 |
| CTRI/2024/09/074175 | 2024 | India | NA | NA | NA | A-PRF | 20 |
| CTRI/2024/09/073715 | 2024 | India | Phase Ⅲ/Ⅳ | Synthetic Bone Graft | NA | NA | 30 |
| CTRI/2024/08/072935 | 2024 | India | NA | NA | NA | Low level Laser Diode and T PRF | 22 |
| CTRI/2024/08/072889 | 2024 | India | Phase Ⅳ | NA | NA | Low level laser therapy | 22 |
| CTRI/2024/08/072547 | 2024 | India | Phase Ⅱ/Ⅲ | Dentinal graft | NA | NA | 30 |
| CTRI/2024/08/072532 | 2024 | India | Phase Ⅲ/Ⅳ | NA | NA | Albumin PRF | 16 |
| CTRI/2024/08/072526 | 2024 | India | Phase Ⅲ | NA | NA | Chitosan | 50 |
| CTRI/2024/08/072462 | 2024 | India | Phase Ⅰ/Ⅱ | Bio-adhesive bone regenerative scaffold | NA | NA | 20 |
| CTRI/2024/08/072377 | 2024 | India | Phase Ⅲ | NA | NA | Local drug delivery of zinc oxide loaded iPrf | 30 |
| CTRI/2024/07/070939 | 2024 | India | NA | NA | NA | PRF | 22 |
| CTRI/2024/06/068965 | 2024 | India | NA | Synthetic Bone Graft | NA | NA | 28 |
| CTRI/2024/06/068727 | 2024 | India | Phase Ⅱ/Ⅲ | NA | NA | Hyaluronic acid/Tetracycline fiber | 128 |
| CTRI/2024/05/068115 | 2024 | India | Phase Ⅳ | Synthetic Bone Graft | Spongy Gelatin Membrane | A three-dimensional bone tissue engineering scaffold with a unique combination of nano-hydroxyapatite, gelatin and carboxymethyl cellulose | 34 |
| CTRI/2024/05/067711 | 2024 | India | Phase Ⅳ | Synthetic Bone Graft | Autologus fibrin glue | NA | 60 |
| CTRI/2024/05/066679 | 2024 | India | Phase Ⅲ | NA | Collagen plug | PRF | 38 |
| CTRI/2024/05/066675 | 2024 | India | Phase Ⅲ | NA | NA | Morinda Citrifolia fruit extract | 44 |
| CTRI/2024/04/066472 | 2024 | India | Phase Ⅳ | Autograft+Xenograft | NA | NA | 36 |
| CTRI/2024/04/065101 | 2024 | India | Phase Ⅳ | Autograft | NA | PRF | 40 |
| CTRI/2024/03/064289 | 2024 | India | Phase Ⅳ | Synthetic Bone Graft | NA | NA | 100 |
| CTRI/2024/03/064054 | 2024 | India | NA | Synthetic bone graft | NA | Simvastatin | 20 |
| CTRI/2024/02/062461 | 2024 | India | Phase Ⅳ | NA | Amniotic membrane | PRF | 48 |
| CTRI/2024/02/062321 | 2024 | India | Phase Ⅳ | Autograft+Xenograft+Bone putty materials | NA | NA | 28 |
| CTRI/2024/01/061503 | 2024 | India | Phase Ⅱ | NA | NA | L-PRF | 20 |
| CTRI/2024/01/061312 | 2024 | India | Phase Ⅲ | NA | NA | PRF/CGF(Sohn’s Poncho technique) | 38 |
| ChiCTR2500096378 | 2024 | China | NA | Synthetic Bone Graft | NA | NA | Group A : 6 Group B : 6 Group C : 6 |
| ChiCTR2400093560 | 2024 | China | NA | NA | NA | The gingival flap is not completely closed and is partially open | Experimental group: 64, Control group: 64. |
| ChiCTR2400092241 | 2024 | China | NA | NA | NA | Sausage technique | Experimental group: 17, Control group: 17. |
| TCTR20231107005 | 2023 | Thailand | Phase Ⅰ | Synthetic Bone Graft | PCL | 3d-printed | 15 |
| TCTR20230601004 | 2023 | NA | Phase Ⅲ | Synthetic Bone Graft | PCL membrane | NA | 64 |
| RBR-10vc6r63 | 2023 | Brazil | NA | NA | NA | A-PRF/L-PRF | 29 |
| PACTR202407576478340 | 2023 | Egypt | NA | NA | NA | Hyaluronic acid | 30 |
| PACTR202402627986765 | 2023 | Egypt | NA | NA | NA | Low level laser | 24 |
| NCT06871657 | 2023 | United States | NA | NA | NA | Sinus lift using osseodensification technique Sinus lift using the osseocondensation technique | 30 |
| NCT06783517 | 2023 | Egypt | NA | Xenograft | NA | Hyaluronic acid | 20 |
| NCT06724783 | 2023 | Switzerland | Phase Ⅳ | NA | Collagen membrane | NA | 68 |
| NCT06625528 | 2023 | Egypt | Phase 0 | Synthetic Bone Graft | NA | PRF | 36 |
| NCT06580626 | 2023 | Egypt | NA | NA | NA | Decompression technique Guided bone regenaration using Ethoss Ridge splitting technqiue | 42 |
| NCT06574815 | 2023 | China | NA | NA | Small intestine submucosa membrane | NA | 36 |
| NCT06523686 | 2023 | Lebanon | Phase Ⅱ/Ⅲ | Autograft+Xenograft | Collagen membrane | NA | 4 |
| NCT06473545 | 2023 | Spain | NA | Synthetic Bone Graft | NA | NA | 173 |
| NCT06444334 | 2023 | Egypt | NA | NA | NA | PRF+low level diode laser | 22 |
| NCT06439264 | 2023 | India | NA | Synthetic Bone Graft | Bentonite clay gel | NA | 24 |
| NCT06426524 | 2023 | India | NA | Synthetic Bone Graft | NA | IGF-1 | 24 |
| NCT06412705 | 2023 | Italy | NA | Xenograft | Collagen membrane | Vit.D3 | 14 |
| NCT06353399 | 2023 | United States | NA | NA | NA | Intra-marrow penetrations | 24 |
| NCT06186232 | 2023 | NA | NA | NA | PMMA membrane | 3d-printed | 6 |
| NCT06172114 | 2023 | NA | NA | Xenograft | NA | L-PRF | 34 |
| NCT06164626 | 2023 | Brazil | Phase Ⅰ | Synthetic Bone Graft | PDO membrane | low level infrared laser therapy (808 nm) | 60 |
| NCT06156488 | 2023 | Spain | NA | Xenograft | NA | Polynucleotide+hyaluronic acid | 5 |
| NCT06061354 | 2023 | Chile | Phase Ⅳ | Xenograft | NA | Teriparatide | 42 |
| NCT06015906 | 2023 | NA | NA | Synthetic Bone Graft | PDO membrane | NA | 150 |
| NCT05971342 | 2023 | NA | NA | NA | Spongy Gelatin Membrane | Apoptotic vesicle | 30 |
| NCT05957926 | 2023 | Egypt | NA | Synthetic Bone Graft | NA | Hyaluronic acid | 20 |
| NCT05937035 | 2023 | Spain | NA | Allograft | NA | NA | 10 |
| NCT05890469 | 2023 | United Kingdom | NA | Xenograft | Collagen membrane | NA | 24 |
| NCT05873673 | 2023 | Egypt | Phase Ⅱ | NA | NA | Coenzyme Q10 | 20 |
| NCT05813340 | 2023 | Egypt | NA | NA | Vascularized inter-positional periosteal connective tissue flap | NA | 24 |
| NCT05601531 | 2023 | Taiwan | NA | Autologous Tooth Root | NA | NA | 10 |
| NCT04367766 | 2023 | Italy | NA | Xenograft | Collagen membrane | NA | 60 |
| KCT0008664 | 2023 | Korea | NA | NA | NA | CGF/PRF | 30 |
| KCT0008393 | 2023 | Korea | NA | Xenograft | NA | NA | 50 |
| jRCTb040230035 | 2023 | Japan | NA | NA | NA | Dental pulp stem cells | 5 |
| IRCT20230609058431N1 | 2023 | Syrian Arab Republic | NA | Xenograft | Collagen membrane | Asigannan | 10 |
| IRCT20230331057791N1 | 2023 | Iran (Islamic Republic of) | Phase Ⅲ | NA | NA | L-PRF | 20 |
| IRCT20221023056272N1 | 2023 | Iran (Islamic Republic of) | Phase Ⅲ | NA | NA | Low-level laser | 22 |
| IRCT20220807055633N2 | 2023 | Iran (Islamic Republic of) | NA | Synthetic Bone Graft | PCL membrane | NA | 7 |
| IRCT20100427003813N13 | 2023 | Iran (Islamic Republic of) | NA | NA | NA | Double valve or periosteal release | 24 |
| DRKS00031642 | 2023 | Germany | NA | Xenograft | Collagen membrane | PRF | 108 |
| DRKS00031607 | 2023 | Germany South Korea | NA | Xenograft/Collagen scaffold | NA | NA | 320 |
| CTRI/2023/12/060979 | 2023 | India | Phase Ⅱ/Ⅲ | NA | Amniotic membrane | NA | 30 |
| CTRI/2023/12/060432 | 2023 | India | Phase Ⅱ/Ⅲ | Synthetic Bone Graft | NA | NA | 40 |
| CTRI/2023/11/059644 | 2023 | India | Phase Ⅱ/Ⅲ | Synthetic Bone Graft | NA | A-PRF | 40 |
| CTRI/2023/10/058270 | 2023 | India | Phase Ⅲ/Ⅳ | NA | NA | CGF+PRF | 30 |
| CTRI/2023/10/058222 | 2023 | India | Phase Ⅱ | Silver Nanoparticles/Titanium nanoparticles | NA | PRF and Chitosan nanoparticles | 90 |
| CTRI/2023/10/058214 | 2023 | India | Phase Ⅲ | NA | NA | Magnesium citrate | 54 |
| CTRI/2023/09/057332 | 2023 | India | NA | Synthetic Bone Graft | NA | NA | 30 |
| CTRI/2023/09/057278 | 2023 | India | Phase Ⅱ | Synthetic Bone Graft | NA | NA | 24 |
| CTRI/2023/08/057033 | 2023 | India | Phase Ⅲ | NA | NA | CGF | 10 |
| CTRI/2023/08/056791 | 2023 | India | Phase Ⅱ | NA | NA | Enamel Matrix derivative | 33 |
| CTRI/2023/08/056460 | 2023 | India | Phase Ⅳ | NA | NA | Hyaluronic acid | 48 |
| CTRI/2023/07/055690 | 2023 | India | Phase Ⅲ | NA | NA | PRF | 70 |
| CTRI/2023/07/055485 | 2023 | India | Phase Ⅳ | NA | NA | Alenophosphate | 22 |
| CTRI/2023/07/055134 | 2023 | India | Phase Ⅳ | NA | NA | Morinda Citrifolia fruit extract | 20 |
| CTRI/2023/07/055045 | 2023 | India | NA | Sticky bone | NA | Simplified Papilla Preservation Technique with Intramarrow Penetration And Low level laser therapy with sticky bone | 40 |
| CTRI/2023/07/054740 | 2023 | India | NA | Xenograft | NA | Low-level laser | 30 |
| CTRI/2023/06/053875 | 2023 | India | Phase Ⅲ/Ⅳ | NA | NA | PRF+Hyaluronic acid | 24 |
| CTRI/2023/06/053742 | 2023 | India | NA | NA | NA | Psidium guajava leaf | 3 |
| CTRI/2023/05/053341 | 2023 | India | Phase Ⅲ/Ⅳ | Dentinal graft | NA | A-PRF+PRF Photobiomodulation | 20 |
| CTRI/2023/04/051757 | 2023 | India | NA | Xenograft | NA | Bone ring technique | 18 |
| CTRI/2023/03/051162 | 2023 | India | Phase Ⅱ/Ⅲ | NA | NA | PRF+Fence technique | 10 |
| CTRI/2023/03/050618 | 2023 | India | NA | NA | NA | Simvastatin | 20 |
| CTRI/2023/02/049568 | 2023 | India | Phase Ⅲ | Synthetic Bone Graft | NA | PRF | 28 |
| CTRI/2023/01/049209 | 2023 | India | NA | Sticky bone | NA | Intramarrow Penetration | 32 |
| CTRI/2023/01/048932 | 2023 | India | Phase Ⅲ | NA | NA | L-PRF | 15 |
| ChiCTR2500097568 | 2023 | China | NA | Allograft | Collagen membrane | NA | Experimental group: 17, Control group: 17. |
| ChiCTR2300075686 | 2023 | China | NA | Synthetic Bone Graft | NA | NA | Experimental group: 78, Control group: 78. |
| ChiCTR2300068228 | 2023 | China | NA | Xenograft | Collagen membrane | NA | Experimental group: 17, Control group: 17. |
| ACTRN12623001002673 | 2023 | Australia | NA | Synthetic Bone Graft | PCL | PRF | 5 |
| TCTR20220510011 | 2022 | Thailand | Phase Ⅳ | Xenograft | NA | PRF | 13 |
| RBR-10m7c788 | 2022 | Brazil | NA | Xenograft | NA | PRF | 18 |
| NCT06615362 | 2022 | Egypt | NA | NA | Dentin-derived membrane | NA | 20 |
| NCT06516523 | 2022 | Brazil | NA | NA | NA | Pulsed electromagnetic field (PEMF) | 80 |
| NCT06378112 | 2022 | Spain | Phase Ⅳ | Xenograft | NA | NA | 40 |
| NCT06312384 | 2022 | Egypt | Phase Ⅲ | NA | NA | 3D Surgical Template on the Contour of Bone Augmentation, in Patient With Labial Alveolar Ridge Defect and Simultaneous Implantation | 20 |
| NCT06059898 | 2022 | Italy Denmark Austria | NA | NA | Resorbable magnesium membrane | NA | 84 |
| NCT06041854 | 2022 | Egypt | NA | Allograft | NA | Enamel Matrix derivative | 20 |
| NCT05743452 | 2022 | China | Phase 0 | Synthetic Bone Graft | NA | 3d-printed | 5 |
| NCT05723094 | 2022 | Malaysia | NA | NA | NA | Ga-Al-As diode laser | 32 |
| NCT05717478 | 2022 | Spain | NA | Xenograft | NA | rhBMP-2 | 40 |
| NCT05658900 | 2022 | Egypt | NA | NA | NA | Albumin PRF | 20 |
| NCT05602493 | 2022 | Spain | Phase Ⅲ | NA | NA | Soludronate | 60 |
| NCT05593198 | 2022 | Italy | NA | NA | NA | Albumin PRF | 44 |
| NCT05577663 | 2022 | Greece | NA | NA | PLGA membrane | NA | 39 |
| NCT05540015 | 2022 | NA | NA | NA | NA | Erbium laser | 60 |
| NCT05505084 | 2022 | United States | NA | NA | Amnioion - chorionic membrane Resorbable collagen membrane | NA | 50 |
| NCT05478421 | 2022 | China | NA | NA | NA | Collagen sponge GBR CS/GBR | 80 |
| NCT05465421 | 2022 | Egypt | NA | NA | NA | Bone marrow mononuclear cells+PRF | 24 |
| NCT05426616 | 2022 | Belgium | NA | NA | TR-d-PTFE | NA | 36 |
| NCT05241548 | 2022 | Egypt | NA | NA | PCL scaffold | BMAC+3d Printed Polycaprolactone (PCL) Scaffold | 7 |
| NCT05219305 | 2022 | United States | NA | Autograft | NA | NA | 36 |
| KCT0007940 | 2022 | Korea | NA | collagen-containing bone block | Collagen membrane | NA | 40 |
| jRCTs032220261 | 2022 | Japan | NA | Synthetic Bone Graft | P（LA/CL）membrane | NA | 20 |
| IRCT20221216056831N1 | 2022 | Iran (Islamic Republic of) | NA | NA | NA | CGF | 45 |
| IRCT20221111056472N1 | 2022 | Iran (Islamic Republic of) | NA | Autograft+Xenograft | Collagen membrane | NA | 15 |
| IRCT20220807055633N1 | 2022 | Iran (Islamic Republic of) | NA | Synthetic Bone Graft | PCL membrane | 3d-printed | 7 |
| IRCT20220407054448N1 | 2022 | Iran (Islamic Republic of) | NA | Allograft | NA | PRF | 20 |
| IRCT20210526051407N2 | 2022 | Iran (Islamic Republic of) | Phase Ⅱ | NA | Self-assembling peptide nanofiber | NA | 12 |
| IRCT20200919048756N5 | 2022 | Iran (Islamic Republic of) | NA | NA | spongy gelatin membrane containing nanocurcumin and nanohydroxyapatite | Curcumin | 20 |
| DRKS00028789 | 2022 | Germany | NA | Synthetic Bone Graft | NA | NA | 54 |
| DRKS00019229 | 2022 | Germany | NA | NA | NA | PRF | 60 |
| CTRI/2022/12/048262 | 2022 | India | Phase Ⅳ | NA | NA | Melatonin | 30 |
| CTRI/2022/11/047795 | 2022 | India | NA | NA | NA | Hyaluronic acid | 32 |
| CTRI/2022/11/047006 | 2022 | India | NA | NA | NA | Mangosteen peel extract gel | 8 |
| CTRI/2022/10/046927 | 2022 | India | Phase Ⅱ | Autologous dentin | NA | A-PRF+PRF+Simvastatin | 12 |
| CTRI/2022/10/046923 | 2022 | India | NA | NA | NA | PRF | 30 |
| CTRI/2022/10/046426 | 2022 | India | NA | Sticky bone | NA | Simvastatin | 44 |
| CTRI/2022/09/045423 | 2022 | India | NA | NA | NA | PRF | 13 |
| CTRI/2022/08/044782 | 2022 | India | NA | NA | Amniotic membrane | L-PRF/Simvastatin | 36 |
| CTRI/2022/08/044750 | 2022 | India | Phase Ⅱ/Ⅲ | NA | NA | Melatonin | 40 |
| CTRI/2022/08/044627 | 2022 | India | Phase Ⅲ | NA | NA | PRF+Rosuvastatin | 52 |
| CTRI/2022/07/044413 | 2022 | India | Phase Ⅱ | NA | NA | Low Level Laser Therapy | 64 |
| CTRI/2022/07/044359 | 2022 | India | Phase Ⅱ | Calcium Sulfate | NA | PRP | 14 |
| CTRI/2022/07/044294 | 2022 | India | NA | NA | Amniotic membrane | Hyaluronic acid | 45 |
| CTRI/2022/07/044008 | 2022 | India | NA | Xenograft | NA | Laser Therapy | 60 |
| CTRI/2022/07/043672 | 2022 | India | NA | NA | NA | PRF/PRP | 20 |
| CTRI/2022/05/042769 | 2022 | India | NA | NA | Collagen membrane | Trans-resveratrol | 10 |
| CTRI/2022/05/042592 | 2022 | India | NA | NA | NA | Lactoferrin microsphere | 52 |
| CTRI/2022/04/041859 | 2022 | India | Phase Ⅲ | Eggshell nano-hydroxyapatite | NA | CGF | 40 |
| CTRI/2022/03/041537 | 2022 | India | Phase Ⅱ/Ⅲ | Eggshell nano-hydroxyapatite | NA | PRF | 52 |
| CTRI/2022/03/041386 | 2022 | India | NA | Eggshell nano-hydroxyapatite | NA | PRF | 20 |
| ChiCTR2300070198 | 2022 | China | NA | NA | NA | The increment of bone inside the arch profile and the increment of bone outside the arch profile | Experimental group: 31, Control group: 31. |
| ChiCTR2300068606 | 2022 | China | NA | Mixed bone meal | Titanium meshes | Sandwich osteotomy | Experimental group: 8, Control group: 8. |
| ChiCTR2200061852 | 2022 | China | NA | Synthetic Bone Graft | NA | NA | Experimental group: 84, Control group: 84. |
| ChiCTR2200058049 | 2022 | China | NA | Synthetic Bone Graft | NA | 3d-printed | Experimental group: 18, Control group: 18. |
| ACTRN12622001002774 | 2022 | Australia | NA | Synthetic Bone Graft | PCL | 3d-printed | 6 |
| ACTRN12622000054718 | 2022 | Australia | NA | NA | NA | Sinus lift | 38 |
| 2021-005511-32 | 2022 | Spain | Phase Ⅲ | NA | NA | Alenophosphate | NA |
| 2021-005374-26 | 2022 | Spain | Phase Ⅲ | Xenograft | NA | L-PRF | NA |
| TCTR20210325002 | 2021 | China | Phase Ⅳ | NA | NA | CGF | 30 |
| RBR-3vn9c5q | 2021 | Brazil | NA | Xenograft | NA | NA | 64 |
| RBR-24mdgrf | 2021 | Dominican Republic | Phase Ⅰ | Autologous dentin | NA | PRP | 140 |
| RBR-2394x96 | 2021 | Brazil | NA | NA | PDO membrane | NA | 36 |
| NL-OMON54923 | 2021 | Netherlands | NA | Synthetic Bone Graft | NA | Stromal vascular fragment | 10 |
| NL-OMON51997 | 2021 | Netherlands | NA | NA | PLGA membrane | Alenophosphate+testosterone | 16 |
| NCT06734962 | 2021 | Latvia | Phase Ⅱ | NA | NA | A-PRF | 44 |
| NCT06627621 | 2021 | Turkey | Phase 0 | NA | E-polycaprolactone membrane | NA | 4 |
| NCT06269497 | 2021 | Norway | NA | TiO2-scaffolds | NA | NA | 10 |
| NCT06139939 | 2021 | Egypt | NA | NA | NA | Double layer technique | 18 |
| NCT06135077 | 2021 | Egypt | NA | Xenograft | NA | T-PRF | 18 |
| NCT06020092 | 2021 | Croatia | Phase Ⅳ | Synthetic Bone Graft | NA | NA | 36 |
| NCT05772975 | 2021 | Bosnia and Herzegovina | Phase Ⅲ | NA | NA | E-PRF/H-PRF | 61 |
| NCT05670067 | 2021 | Egypt | NA | Autograft+Xenograft | NA | NA | 24 |
| NCT05475730 | 2021 | Italy | NA | Xenograft | NA | Hyaluronic acid | 5 |
| NCT05311735 | 2021 | United States | NA | Autologous dentin | NA | NA | 45 |
| NCT05279911 | 2021 | Egypt | NA | NA | NA | Low-level laser therapy (LLLT) | 40 |
| NCT05122299 | 2021 | Egypt | NA | NA | NA | Coenzyme Q10 | 18 |
| NCT05100615 | 2021 | Egypt | NA | Synthetic Bone Graft | NA | PRF | 24 |
| NCT05013580 | 2021 | United States | NA | NA | Amnioion - chorionic membrane | NA | 20 |
| NCT05010785 | 2021 | Egypt | NA | NA | NA | PRF | 22 |
| NCT04998058 | 2021 | Brazil | Phase Ⅰ/Ⅱ | Synthetic Bone Graft | Collagen membrane | NA | 20 |
| NCT04959513 | 2021 | Belgium | NA | NA | NA | L-PRF | 15 |
| NCT04937023 | 2021 | India | NA | NA | NA | Ursodeoxycholic acid | 26 |
| NCT04932161 | 2021 | India | NA | Titanium Granules | NA | NA | 12 |
| NCT04886947 | 2021 | NA | NA | NA | NA | Cord stem cell | 20 |
| NCT04843488 | 2021 | Hungary | NA | NA | PTFE+collagen membrane | NA | 30 |
| NCT04835532 | 2021 | China | NA | Xenograft | Collagen membrane | A-PRF+PRF | 69 |
| NCT04789759 | 2021 | NA | NA | Synthetic Bone Graft | NA | NA | 20 |
| NCT04773847 | 2021 | NA | NA | Synthetic Bone Graft | NA | 3d-printed | 20 |
| NCT04462575 | 2021 | Egypt | NA | NA | Collagen membrane | NA | 30 |
| NCT04440241 | 2021 | Spain | NA | Xenograft | Collagen membrane | Submerged healing | 44 |
| NCT03678467 | 2021 | United States | Phase Ⅰ/Ⅱ | NA | NA | Stem cells+growth factor | 6 |
| KCT0006428 | 2021 | Korea | Phase Ⅳ | Synthetic Bone Graft | Collagen membrane | NA | 60 |
| KCT0005732 | 2021 | Korea | Phase Ⅰ/Ⅱ | Ceramic scaffold | NA | 3d-printed | 60 |
| jRCT2061210032 | 2021 | Japan | Phase Ⅰ/Ⅱ | Synthetic Bone Graft | NA | OIF | 8 |
| jRCT2032210306 | 2021 | Japan | Phase Ⅲ | Collagen-based bone regeneration material | NA | NA | 24 |
| IRCT20230714058773N1 | 2021 | Pakistan | NA | NA | NA | PRF | 60 |
| IRCT20200919048756N2 | 2021 | Iran (Islamic Republic of) | NA | NA | Collagen membrane | Curcumin+Aspiri | 10 |
| DRKS00020222 | 2021 | Spain Switzerland | Phase Ⅳ | Synthetic Bone Graft | Collagen membrane | NA | 64 |
| CTRI/2021/09/036778 | 2021 | India | NA | MTA | NA | PRF | 32 |
| CTRI/2021/09/036533 | 2021 | India | Phase Ⅱ | Synthetic Bone Graft | NA | PRF | 12 |
| CTRI/2021/07/035169 | 2021 | India | NA | NA | NA | Metformin | 20 |
| CTRI/2021/06/033999 | 2021 | India | NA | Autograft | NA | NA | 45 |
| CTRI/2021/04/033061 | 2021 | India | NA | Synthetic Bone Graft | NA | PRF | 30 |
| CTRI/2021/04/032737 | 2021 | India | Phase Ⅱ | Synthetic Bone Graft | NA | NA | 20 |
| CTRI/2021/04/032486 | 2021 | India | Phase Ⅱ/Ⅲ | Synthetic Bone Graft | NA | Saline hydration or blood hydration | 20 |
| CTRI/2021/02/031513 | 2021 | India | Phase Ⅳ | NA | NA | Simvastatin | 30 |
| CTRI/2021/02/030978 | 2021 | India | NA | Allograft/Synthetic Bone Graft | NA | NA | 38 |
| UMIN000047625 | 2020 | Japan | NA | Synthetic Bone Graft+xenograft | NA | NA | 30 |
| TCTR20200526010 | 2020 | Thailand | Phase Ⅱ | NA | PCL membrane | NA | 24 |
| RBR-9wqkr5c | 2020 | Dominican Republic | NA | Biomaterial scaffold | NA | 3d-printed | 15 |
| RBR-523n7r | 2020 | Brazil | NA | NA | NA | Simvastatin | 22 |
| RBR-3csg3j | 2020 | Brazil | NA | Autograft+Xenograft | TR-d-PTFE/resorbable xenogenic collagen membrane | NA | 17 |
| RBR-3bjk6c | 2020 | Brazil | Phase Ⅰ/Ⅱ | Xenograft | NA | L-PRF | 11 |
| RBR-2spcx5 | 2020 | Brazil | Phase Ⅳ | NA | NA | Laser Therapy | 34 |
| NCT06334159 | 2020 | Spain | NA | NA | Collagen membrane | NA | 26 |
| NCT05020405 | 2020 | Egypt | NA | NA | NA | Simvastatin | 22 |
| NCT04945629 | 2020 | Spain | Phase Ⅲ | NA | NA | PRGF | 44 |
| NCT04679766 | 2020 | NA | NA | NA | NA | Ice cream cone technique with immediate implant as a flapless bone regenerative method in management of patients with labial plte dehiscence | 10 |
| NCT04376320 | 2020 | Egypt | NA | NA | NA | customised ceramic sheets (Group 1) modified sausage technique (Group 2) | 14 |
| NCT04338139 | 2020 | Lebanon | NA | Xenograft | NA | NA | 30 |
| NCT04297813 | 2020 | Spain France Denmark Norway | Phase Ⅲ | Xenograft | NA | Mesenchymal stem cells | 150 |
| NCT04257097 | 2020 | Italy | NA | NA | TR-PTFE | NA | 50 |
| KCT0005348 | 2020 | Korea | NA | Block type collagenated bone | NA | NA | 70 |
| KCT0004906 | 2020 | Korea | NA | Xenograft | NA | NA | 30 |
| jRCTs072200063 | 2020 | Japan | Phase Ⅰ/Ⅱ | NA | PLCL membrane | NA | 20 |
| jRCTs032200108 | 2020 | Japan | Phase Ⅰ | Collagenated bone | NA | NA | 4 |
| jRCTc020190025 | 2020 | Japan | NA | MTA | NA | CGF | 24 |
| jRCTb070190059 | 2020 | Japan | NA | Xenograft | NA | Non-cultured autologous adipose tissue-derived stem/progenitor cells+rhBMP-2 | 15 |
| jRCTb030190222 | 2020 | Japan | NA | NA | NA | Bone marrow stromal cells | 10 |
| IRCT20190114042354N1 | 2020 | Iran (Islamic Republic of) | NA | Allograft | NA | PRF | 20 |
| DRKS00036005 | 2020 | Germany Poland | NA | Xenograft | NA | PRF | 64 |
| DRKS00029128 | 2020 | India | NA | NA | NA | PRF/Cissus quadrangularis | 60 |
| DRKS00018875 | 2020 | Belgium Switzerland | Phase Ⅳ | NA | NA | Arm 1：Ridge preservation (placement of the implant 5 months after tooth extraction and filling with biomaterials) Arm 2：Early Implant Placement (Placement of the implant 2 months after tooth extraction and together with biomaterials) | 47 |
| CTRI/2020/12/029775 | 2020 | India | NA | Sticky bone | NA | NA | 19 |
| CTRI/2020/11/028962 | 2020 | India | NA | NA | NA | A-PRF/L-PRF | 26 |
| CTRI/2020/11/028865 | 2020 | India | Phase Ⅰ/Ⅱ | NA | NA | PRF | 100 |
| CTRI/2020/11/028807 | 2020 | India | NA | Synthetic Bone Graft | PGLA membrane | NA | 12 |
| CTRI/2020/10/028467 | 2020 | India | Phase Ⅰ | Autograft | NA | NA | 30 |
| CTRI/2020/07/026597 | 2020 | India | NA | Sticky bone | Chorion | NA | 38 |
| CTRI/2020/06/025567 | 2020 | India | Phase Ⅰ | Sticky bone | Collagen membrane | NA | 36 |
| CTRI/2020/04/024771 | 2020 | India | Phase Ⅲ | NA | NA | Simvastatin | 40 |
| CTRI/2020/03/024076 | 2020 | India | Phase Ⅰ | NA | Collagen membrane | Oleuropein | 20 |
| CTRI/2020/03/024069 | 2020 | India | Phase Ⅰ | NA | Chorion | Astaxanthin | 20 |
| CTRI/2020/03/023751 | 2020 | India | NA | Sticky bone | NA | NA | 24 |
| CTRI/2020/02/023646 | 2020 | India | Phase Ⅰ/Ⅱ | Autograft+Xenograft | NA | NA | 20 |
| ChiCTR2000034211 | 2020 | China | NA | NA | NA | GLP-1 | Case series : 400 |
| ChiCTR2000030212 | 2020 | China | NA | NA | Collagen membrane | NA | Experimental group: 98, Control group: 98. |
| NCT06420713 | 2019 | Brazil | NA | Autograft+Xenograft | NA | L-PRF | 28 |
| NCT06154057 | 2019 | Brazil | NA | Synthetic Bone Graft | NA | NA | 100 |
| NCT05990283 | 2019 | Turkey | NA | NA | NA | Socket shield technique | 24 |
| NCT05662709 | 2019 | Spain | Phase Ⅲ | NA | NA | L-PRF | 28 |
| NCT05538715 | 2019 | Hungary | Phase Ⅳ | NA | NA | Split-thickness group Full-thickness group | 44 |
| NCT05445986 | 2019 | China | NA | NA | NA | NA | 29 |
| NCT05282667 | 2019 | Spain | NA | Bone graft | Collagen membrane | NA | 38 |
| NCT05264480 | 2019 | Hungary | NA | NA | NA | Bone augmentation with minimally invasive corticotomy (piezotomy) | 20 |
| NCT04816110 | 2019 | Egypt | Phase Ⅱ | NA | collagen membrane | PRP+PRF | 16 |
| NCT04763187 | 2019 | Lithuania | NA | NA | NA | PRGF/PRF | 43 |
| NCT04514991 | 2019 | United States | NA | Collagenated bone | NA | NA | 67 |
| NCT04232202 | 2019 | Slovenia | NA | NA | NA | Er:YAG laser LightWalker (Fotona) Nd:YAG laser Twinlight photobiomodulation | 29 |
| NCT04197128 | 2019 | India | NA | NA | Ribose cross linked collagen membrane | Ribose | 28 |
| NCT04141215 | 2019 | France | NA | Allograft | NA | NA | 86 |
| NCT04093583 | 2019 | Spain | Phase Ⅲ | NA | NA | PRGF | 46 |
| NCT04022538 | 2019 | NA | NA | NA | NA | Anterior maxillary vertical segmental sandwich osteotomy with simultaneous implant placement Anterior maxillary vertical segmental sandwich osteotomy using micro-plates fixation | 20 |
| NCT04013425 | 2019 | Egypt | NA | NA | NA | Ice Cream Cone Technique | 20 |
| NCT04001842 | 2019 | Egypt | NA | Axially vascularized bone substitute | NA | NA | 10 |
| NCT03985033 | 2019 | Hong Kong | NA | NA | NA | L-PRF | 18 |
| NCT03978962 | 2019 | France | NA | NA | Collagen membrane | NA | 56 |
| NCT03900741 | 2019 | Spain | NA | NA | NA | Submerged healing Non-submerged healing | 36 |
| NCT03895060 | 2019 | NA | NA | Autograft | Collagen membrane | NA | 10 |
| NCT03855852 | 2019 | Egypt | NA | Xenograft | NA | A-PRF | 20 |
| NCT03840681 | 2019 | NA | NA | Xenograft | TR-PTFE | NA | 20 |
| NCT03839615 | 2019 | Egypt | NA | Autograft+Xenograft | PTFE | NA | 10 |
| NCT03656484 | 2019 | Romania | Phase Ⅱ | NA | NA | Hyaluronic acid+melatonin | 50 |
| KCT0004014 | 2019 | Korea | NA | Xenograft | Collagen membrane | NA | 18 |
| jRCTs072190012 | 2019 | Japan | Phase Ⅰ | NA | PLCL membrane | NA | 5 |
| jRCTs052180215 | 2019 | Japan | Phase Ⅰ | NA | Titanium plate | Laser Therapy | 24 |
| jRCTb041190076 | 2019 | Japan | NA | Synthetic Bone Graft | NA | Bone marrow-derived mesenchymal cells+PRP | 29 |
| IRCT20181205041860N1 | 2019 | Iran (Islamic Republic of) | NA | NA | NA | AT-NMCs | 20 |
| CTRI/2019/10/021714 | 2019 | India | Phase Ⅳ | Autograft | NA | PRF | 42 |
| CTRI/2019/09/021017 | 2019 | India | Phase Ⅲ | Eggshell derived calcium phosphate bone cement | NA | NA | 30 |
| CTRI/2019/07/020377 | 2019 | India | NA | Synthetic Bone Graft | NA | PRF | 80 |
| CTRI/2019/07/020259 | 2019 | India | NA | NA | NA | PRF | 65 |
| CTRI/2019/03/018203 | 2019 | India | NA | NA | Titanium mesh | NA | 12 |
| CTRI/2019/02/017578 | 2019 | India | Phase Ⅰ | NA | NA | PRF+Aspiri | 60 |
| CTRI/2019/01/017101 | 2019 | India | NA | NA | NA | PRF | 50 |
| ACTRN12619001124123 | 2019 | Australia | NA | Synthetic Bone Graft+xenograft | NA | NA | 12 |
| 2018-001227-39 | 2019 | Denmark Norway Spain | Phase Ⅱ/Ⅲ | Synthetic Bone Graft | NA | mesenchymal stem cells | 150 |
| UMIN000033847 | 2018 | Japan | Phase Ⅰ/Ⅱ | NA | NA | Bone marrow stromal cells | 3 |
| UMIN000032549 | 2018 | Japan | NA | NA | Titanium retina | Laser Therapy | 10 |
| TCTR20181217004 | 2018 | NA | Phase Ⅱ | NA | NA | Acemannan | 30 |
| TCTR20181011001 | 2018 | Thailand | NA | Autograft | NA | PRF | 13 |
| TCTR20180112002 | 2018 | Thailand | NA | NA | NA | PRF | 40 |
| NCT06317090 | 2018 | Belgium | NA | Autograft+Xenograft | NA | L-PRF | 6 |
| NCT06226116 | 2018 | Spain | NA | Autologous dentin | NA | NA | 27 |
| NCT05975892 | 2018 | Venezuela | Phase Ⅰ/Ⅱ | NA | NA | Bone marrow mesenchymal stromal cells | 30 |
| NCT05791123 | 2018 | Egypt | NA | Xenograft | NA | PRF | 14 |
| NCT04480073 | 2018 | Italy | NA | NA | NA | NA | 24 |
| NCT04451486 | 2018 | Taiwan | Phase Ⅰ | NA | NA | Peripheral blood stem cell | 24 |
| NCT04443972 | 2018 | Egypt | NA | Synthetic Bone Graft | Collagen membrane | NA | 14 |
| NCT04329351 | 2018 | Brazil | NA | NA | PTFE-d | NA | 40 |
| NCT03731416 | 2018 | NA | NA | Autograft+Xenograft | Collagen membrane | NA | 10 |
| NCT03712631 | 2018 | NA | NA | Autograft+Xenograft | NA | Tunneling technique | 50 |
| NCT03697616 | 2018 | NA | NA | Sticky bone | NA | CGF | 24 |
| NCT03680118 | 2018 | NA | NA | Autograft | Collagen membrane | NA | 24 |
| NCT03635658 | 2018 | NA | NA | NA | Titanium mesh/collagen membrane | NA | 20 |
| NCT03585751 | 2018 | NA | NA | NA | NA | Simvastatin+Antibiotic | 45 |
| NCT03584997 | 2018 | NA | NA | Autograft+Xenograft | NA | PRF | 20 |
| NCT03584984 | 2018 | NA | NA | Autograft+Xenograft | Spongy Gelatin Membrane | NA | 30 |
| NCT03573193 | 2018 | NA | NA | Xenograft/Synthetic Bone Graft | NA | NA | 20 |
| NCT03495700 | 2018 | Belgium | NA | Xenograft | NA | L-PRF | 24 |
| NCT03466840 | 2018 | Egypt | NA | NA | Titanium mesh | The coronally advanced lingual flap  Modified periosteal releasing Incision | 7 |
| NCT03071523 | 2018 | Egypt | NA | NA | NA | periosteal releasing incision | 14 |
| KCT0003004 | 2018 | Korea | NA | NA | ePTFE membrane | NA | 25 |
| ISRCTN11458658 | 2018 | Portugal | NA | Autograft | NA | NA | 56 |
| IRCT20201012049004N1 | 2018 | Iran (Islamic Republic of) | NA | Xenograft | Amniotic membrane | NA | 20 |
| IRCT20171108037316N1 | 2018 | Iran (Islamic Republic of) | Phase Ⅱ/Ⅲ | NA | NA | PRP | 26 |
| DRKS00014324 | 2018 | Brazil | NA | Xenograft | Collagen membrane | Preserve part of the root | 40 |
| DRKS00014049 | 2018 | Germany | NA | Synthetic Bone Graft | NA | NA | 95 |
| CTRI/2018/11/016450 | 2018 | India | Phase Ⅰ | Allograft | NA | PRF | 10 |
| CTRI/2018/10/015965 | 2018 | India | Phase Ⅳ | NA | NA | CGF/PRF | 36 |
| CTRI/2018/07/014897 | 2018 | India | Phase Ⅳ | NA | NA | Curcumin | 10 |
| CTRI/2018/06/014597 | 2018 | India | Phase Ⅱ | GBR | NA | NA | 32 |
| CTRI/2018/05/014221 | 2018 | India | Phase Ⅲ/Ⅳ | NA | NA | PRF+Ornidazole | 20 |
| CTRI/2018/05/013707 | 2018 | India | Phase Ⅰ | NA | NA | LIPUS Open flap debridement | 40 |
| CTRI/2018/04/013497 | 2018 | India | NA | Autograft+Xenograft | Collagen membrane | NA | 12 |
| CTRI/2018/04/012953 | 2018 | India | NA | Allograft | NA | Low level laser therapy | 10 |
| CTRI/2018/02/011881 | 2018 | India | NA | Xenograft | NA | Alenophosphate sodium/metformin | 12 |
| CTRI/2018/01/011302 | 2018 | India | Phase Ⅳ | Synthetic Bone Graft | NA | NA | 40 |
| ChiCTR1800016938 | 2018 | China | NA | GBR | NA | NA | Experimental group: 60, Control group: 60. |
| ACTRN12618000323224 | 2018 | Australia | NA | Synthetic Bone Graft | NA | NA | 20 |
| UMIN000028915 | 2017 | Japan | NA | Synthetic Bone Graft | PLA mesh plate | NA | 15 |
| UMIN000027268 | 2017 | Japan | Phase Ⅰ | NA | NA | Adipocyte | 15 |
| TCTR20171010005 | 2017 | Thailand | Phase Ⅱ | Allograft | Polyethylene membrane | NA | 15 |
| RBR-95m73t | 2017 | Brazil | NA | Xenograft | NA | L-PRF | 24 |
| RBR-56q7h9 | 2017 | Brazil | NA | NA | NA | Grape seed and grapefruit seed extract | 30 |
| NCT04709523 | 2017 | NA | NA | xenograft | NA | PRF | 40 |
| NCT04376060 | 2017 | Lebanon | NA | Xenograft | NA | NA | 15 |
| NCT04286334 | 2017 | Italy | NA | NA | Titanium mesh with collagen membrane | 3d-printed | 30 |
| NCT04149080 | 2017 | Brazil | NA | NA | Polypropylene membrane | Simvastatin | 30 |
| NCT03897010 | 2017 | Egypt | Phase Ⅳ | Synthetic Bone Graft | NA | NA | 5 |
| NCT03646734 | 2017 | NA | NA | Granular bone/block bone | NA | NA | 30 |
| NCT03468998 | 2017 | United States | NA | Allograft | NA | NA | 37 |
| NCT03417375 | 2017 | United States | NA | Allograft | NA | NA | 11 |
| NCT03137979 | 2017 | China | Phase Ⅰ/Ⅱ | NA | NA | Dental pulp stem cells | 30 |
| NCT03076138 | 2017 | Russian Federation | NA | Synthetic Bone Graft | NA | Ribose | 20 |
| NCT03065803 | 2017 | NA | NA | NA | NA | Buccal plate expansion technique | 20 |
| NCT02387008 | 2017 | United States | NA | Allograft | PLA | NA | 6 |
| IRCT20210515051308N1 | 2017 | Iran (Islamic Republic of) | NA | Allograft | NA | NA | 22 |
| DRKS00027567 | 2017 | Syria | NA | Xenograft | TR-d-PTFE | A-PRF | 20 |
| CTRI/2017/12/010949 | 2017 | India | NA | NA | NA | PRF | 150 |
| CTRI/2017/11/010492 | 2017 | India | Phase Ⅳ | Synthetic Bone Graft | NA | Curcumin | 60 |
| CTRI/2017/11/010432 | 2017 | India | Phase Ⅰ | Autograft | NA | NA | 10 |
| CTRI/2017/10/010266 | 2017 | India | NA | Synthetic Bone Graft | NA | rhPDGF-BB | 20 |
| CTRI/2017/06/008881 | 2017 | India | NA | NA | NA | PRF+Simvastatin | 33 |
| ChiCTR-IOR-17013061 | 2017 | China | NA | Synthetic Bone Graft | NA | NA | Experimental group: 24, Control group: 24. |
| ACTRN12617001279314 | 2017 | Australia | Phase Ⅰ | Synthetic Bone Graft | NA | NA | 10 |
| UMIN000020398 | 2016 | Japan | Phase Ⅱ | Synthetic Bone Graft | NA | Bone marrow-derived mesenchymal cells+PRP | 4 |
| PACTR201512001348246 | 2016 | Egypt | NA | Synthetic Bone Graft | NA | NA | 18 |
| NL-OMON42402 | 2016 | Netherlands | NA | Synthetic Bone Graft | PLGA membrane | NA | 11 |
| NCT05753917 | 2016 | NA | NA | NA | NA | Piezoelectric operation | 6 |
| NCT04744012 | 2016 | NA | NA | Autograft+Xenograft | NA | PRGF | 8 |
| NCT04299750 | 2016 | Italy | NA | NA | NA | NA | 32 |
| NCT04131894 | 2016 | NA | Phase Ⅳ | Autologous dentin | NA | PRF | 9 |
| NCT03851289 | 2016 | NA | NA | Calcium sulfate | NA | PRF | 10 |
| NCT03787342 | 2016 | Egypt | NA | NA | Titanium mesh | Double Flap Incision Modified Periosteal Releasing Incision Coronally Advanced Lingual Flap Periosteal releasing incision | 38 |
| NCT03785717 | 2016 | Colombia | NA | NA | NA | Shock Wave | 28 |
| NCT03497403 | 2016 | United States | NA | NA | NA | NA | 30 |
| NCT03419429 | 2016 | NA | Phase Ⅳ | NA | Modified perforated membrane | Simvastatin | 40 |
| NCT03166475 | 2016 | NA | NA | NA | NA | Alveolar ridge preservation | 30 |
| NCT03028038 | 2016 | Syrian Arab Republic | Phase Ⅰ | NA | NA | PRF | 16 |
| NCT03003013 | 2016 | Egypt | Phase Ⅳ | Synthetic Bone Graft | NA | PRF | 28 |
| NCT02985645 | 2016 | Israel | NA | NA | NA | CGF | 20 |
| NCT02890680 | 2016 | Brazil | NA | NA | NA | PRF | 20 |
| NCT02844569 | 2016 | United States | NA | Xenograft | Collagen membrane | 3d-printed | 24 |
| NCT02842619 | 2016 | Israel | Phase Ⅰ/Ⅱ | Synthetic Bone Graft | NA | AT-MSCs | 20 |
| NCT02155764 | 2016 | Russian Federation | Phase Ⅱ | Synthetic Bone Graft | NA | NA | 60 |
| KCT0001814 | 2016 | Korea | Phase Ⅳ | NA | Collagen membrane | NA | 58 |
| IRCT2016022624622N2 | 2016 | Iran (Islamic Republic of) | NA | Synthetic Bone Graft | NA | NA | 100 |
| IRCT2016022526769N1 | 2016 | Iran (Islamic Republic of) | Phase Ⅰ/Ⅱ | NA | NA | Pulsed electromagnetic field | 32 |
| UMIN000018192 | 2015 | Japan | NA | Synthetic Bone Graft | NA | NA | 60 |
| UMIN000016515 | 2015 | Japan | NA | NA | NA | Dental pulp stem cells | 10 |
| NCT04803500 | 2015 | NA | Phase Ⅱ | NA | NA | Simvastatin | 10 |
| NCT03391258 | 2015 | Portugal | NA | NA | NA | L-PRF | 16 |
| NCT02741752 | 2015 | NA | NA | NA | NA | Cortical perforation | 14 |
| NCT02703480 | 2015 | United States | NA | NA | d-PTFE Ti-mesh | NA | 11 |
| KCT0001709 | 2015 | Korea | NA | Xenograft | Collagen membrane | NA | 30 |
| ISRCTN86409051 | 2015 | Türkiye | NA | Xenograft | NA | PRF | 27 |
| ISRCTN14620180 | 2015 | Mexico | NA | Synthetic Bone Graft | NA | PRGF | 10 |
| IRCT2015102024622N1 | 2015 | Iran (Islamic Republic of) | NA | Titanium Granules | NA | NA | 50 |
| DRKS00009496 | 2015 | Switzerland | NA | Xenograft | Collagen membrane | NA | 38 |
| CTRI/2015/03/005636 | 2015 | India | Phase Ⅱ/Ⅲ | Synthetic Bone Graft | NA | Low level Laser | 30 |
| ChiCTR-IOR-15006842 | 2015 | China | NA | Autograft+Xenograft | Collagen membrane | NA | Experimental group: 30, Control group: 30. |
| ChiCTR1800015644 | 2015 | China | NA | NA | NA | Bone marrow stem cells concentrate | Experimental group: 10, Control group: 10. |
| ACTRN12615001013550 | 2015 | NA | NA | NA | NA | Low level laser | 46 |
| ACTRN12615000027516 | 2015 | Australia | NA | NA | Collagen membrane | NA | 20 |
| UMIN000041012 | 2014 | Japan | NA | NA | PGLA membrane | NA | 18 |
| UMIN000019783 | 2014 | Japan | NA | Titanium Granules | NA | NA | 100 |
| UMIN000015206 | 2014 | Japan | NA | NA | Titanium mesh | NA | 10 |
| TCTR20140311001 | 2014 | Thailand | NA | Xenograft | NA | PRF | 20 |
| PACTR201509000914264 | 2014 | Egypt | NA | NA | Collagen membrane | PRF | 20 |
| NCT04331028 | 2014 | Spain | NA | NA | NA | Shockwave therapy | NA |
| NCT04109417 | 2014 | Egypt | NA | NA | NA | PRP | 15 |
| NCT02639572 | 2014 | India | Phase Ⅱ | Synthetic Bone Graft | NA | NA | 30 |
| NCT02583737 | 2014 | NA | NA | Allograft | NA | NA | 15 |
| NCT02396056 | 2014 | United States | NA | NA | Collagen membrane | NA | 10 |
| NCT02330523 | 2014 | United States | NA | Allograft/Xenograft | NA | NA | 40 |
| ISRCTN11165901 | 2014 | Argentina | NA | Allograft | NA | NA | 15 |
| DRKS00005803 | 2014 | Switzerland | NA | Xenograft | NA | NA | 29 |
| CTRI/2014/12/005340 | 2014 | India | NA | Eggshell hydroxyapatite | NA | NA | 100 |
| CTRI/2014/12/005237 | 2014 | India | Phase Ⅳ | NA | Collagen membrane | Diode Laser | 40 |
| CTRI/2014/09/004947 | 2014 | India | NA | Eggshell hydroxyapatite | NA | NA | 20 |
| CTRI/2014/06/004684 | 2014 | India | Phase Ⅱ/Ⅲ | NA | NA | PRF | 40 |
| CTRI/2014/05/004578 | 2014 | India | NA | Synthetic Bone Graft/Xenograft | NA | NA | 24 |
| UMIN000045309 | 2013 | Japan | NA | NA | NA | Bone marrow stromal cells | 8 |
| UMIN000011290 | 2013 | Japan | NA | NA | NA | Bone marrow mesenchymal stem cells | 20 |
| PACTR201307000584171 | 2013 | Egypt | NA | NA | Titanium meshes | NA | 14 |
| NL-OMON38655 | 2013 | Netherlands | NA | Autograft | NA | Improved slow drilling scheme | 30 |
| NCT04942223 | 2013 | NA | NA | Custom mesh guides bone regeneration | NA | NA | 20 |
| NCT04332679 | 2013 | Italy | NA | NA | Titanium meshes | NA | 40 |
| NCT03946020 | 2013 | NA | NA | Autograft+Xenograft | NA | NA | 14 |
| NCT03879967 | 2013 | Switzerland | NA | Allograft | NA | NA | 13 |
| NCT03302143 | 2013 | NA | NA | Xenograft | NA | NA | 48 |
| NCT03290638 | 2013 | NA | NA | NA | Amnioion - chorionic membrane | NA | 43 |
| NCT02373787 | 2013 | Germany | NA | NA | Collagen membrane | NA | 49 |
| NCT01932164 | 2013 | Brazil | NA | NA | NA | Mesenchymal stem cells | 5 |
| NCT01878084 | 2013 | Egypt | NA | Synthetic Bone Graft | NA | NA | 9 |
| NCT01654627 | 2013 | Israel | Phase Ⅰ/Ⅱ | NA | Ammonio Methacrylate Copolymer Type A | NA | 32 |
| CTRI/2013/01/003286 | 2013 | India | NA | NA | NA | Alenophosphate | 30 |
| ChiCTR2000040551 | 2013 | China | NA | Xenograft | Collagen membrane | Different notch design | Group A : 30 Group B : 30 Group C : 30 |
| UMIN000012596 | 2012 | Japan | NA | Autograft | NA | PRP | 30 |
| NCT03432702 | 2012 | Peru | NA | Autograft+Xenograft | NA | NA | 42 |
| NCT02602223 | 2012 | NA | Phase Ⅱ | NA | Amnioion - chorionic membrane | NA | 9 |
| NCT01628367 | 2012 | United States | Phase Ⅳ | NA | NA | Cytoplasmic technique | 32 |
| NCT01616953 | 2012 | United States | Phase Ⅰ/Ⅱ | Autograft | NA | Lxmyelocel-T | 18 |
| NCT01603693 | 2012 | Israel | NA | Synthetic Bone Graft+Xenograft | NA | NA | 13 |
| NCT01572298 | 2012 | United States | NA | Allograft+Autograft | NA | NA | 34 |
| IRCT201203047949N2 | 2012 | Iran (Islamic Republic of) | NA | Allograft | NA | NA | 16 |
| UMIN000041510 | 2011 | Japan | NA | Autograft | NA | NA | 40 |
| UMIN000011286 | 2011 | Japan | NA | NA | NA | Growth factors secreted by the patient's own stem cells | 10 |
| UMIN000005919 | 2011 | Japan | NA | Synthetic Bone Graft | NA | FGF-2 | 20 |
| NL-OMON41609 | 2011 | Netherlands | NA | Synthetic Bone Graft+autograft | Polyethylene glycol | NA | 75 |
| NCT03179683 | 2011 | NA | NA | NA | NA | Diode laser application | 12 |
| NCT01389661 | 2011 | Spain | Phase Ⅰ/Ⅱ | NA | NA | Bone marrow mesenchymal stem cells | 11 |
| NCT01357785 | 2011 | China | Phase Ⅰ | NA | NA | periodontal ligament stem cells | 35 |
| NCT01279187 | 2011 | United States | Phase Ⅱ | NA | NA | Teriparatide | 27 |
| UMIN000003778 | 2010 | Japan | NA | NA | NA | CGF | 10 |
| NL-OMON36465 | 2010 | Netherlands | Phase Ⅲ | Titanium Granules | NA | NA | 12 |
| NCT02755922 | 2010 | NA | Phase Ⅲ | NA | NA | mesenchymal stem cells | 20 |
| NCT01107600 | 2010 | Italy | NA | Xenograft | NA | NA | NA |
| DRKS00005974 | 2010 | Switzerland | NA | NA | ePTFE membrane/Resorbable collagen membrane | NA | 27 |
| NCT01147315 | 2009 | France | NA | Synthetic Bone Graft+autograft | NA | NA | 13 |
| NCT01012921 | 2009 | Belgium Spain Switzerland Italy Germany Hungary Sweden | NA | NA | Polyethylene glycol/Resorbable collagen membrane | NA | 117 |
| NCT00889265 | 2009 | United States | NA | Allograft | Pericardium | NA | 51 |
| NL-OMON30182 | 2008 | Netherlands | NA | Synthetic Bone Graft+autograft | NA | NA | 20 |
| NCT02500654 | 2008 | Sweden | Phase Ⅳ | NA | NA | Enamel matrix derivatives | 31 |
| NCT01105026 | 2008 | NA | Phase Ⅰ | Synthetic Bone Graft | NA | NA | 14 |
| NCT00755911 | 2008 | United States | Phase Ⅰ/Ⅱ | NA | NA | Tissue Repair Cells (TRC) | 24 |
| NCT00639860 | 2008 | United States | Phase Ⅰ/Ⅱ | NA | Collagen membrane | NA | 10 |
| NCT00836797 | 2007 | India Singapore | NA | NA | PLGA scaffold | NA | 33 |
| NCT00454038 | 2007 | Israel | Phase Ⅰ/Ⅱ | NA | Collagen membrane | NA | 100 |
| 2006-003551-20 | 2007 | Austria | Phase Ⅱ | Xenograft | NA | Erythropoietin | NA |
| NCT00727818 | 2006 | Switzerland | Phase Ⅳ | NA | Biongtr™ biodegradable membrane | NA | NA |
| NCT00901121 | 2005 | Italy | NA | Synthetic Bone Graft | NA | NA | 48 |
| 2005-001885-14 | 2005 | Belgium | Phase Ⅱ | NA | NA | Recombinant soluble human tissue factor | NA |
| UMIN000006720 | 2001 | Japan | NA | Transplantation of tissue engineered bone | NA | NA | 100 |

Other: Refers to drugs, growth factors, cell therapies, laser treatments, and surgical methods used for bone regeneration.

NA: Not applicable.
